# Compact and Real-Time Radiation Dosimeter Using Silicon Photomultipliers for In Vivo Dosimetry in Radiation Therapy

**DOI:** 10.3390/s25030857

**Published:** 2025-01-31

**Authors:** Jeongho Kim, Jeehoon Park, Byungdo Park, Yonghoon Kim, Beomjun Park, So Hyun Park

**Affiliations:** 1 Department of Radiation Oncology, Samsung Changwon Hospital, Sungkyunkwan University School of Medicine, Changwon 51353, Republic of Korea; jeongho5248@gmail.com (J.K.); honesty08@daum.net (J.P.); byungdo.park@gmail.com (B.P.); 2 Marine Radioactivity Monitoring Group, Korea Marine Environment Management Co., Busan 48931, Republic of Korea; 3 Interdisciplinary Program in Precision Public Health, Korea University, Seoul 02841, Republic of Korea; 4 Department of Chemistry, Konkuk University, Seoul 05029, Republic of Korea; 5 Department of Radiation Oncology, Jeju National University Hospital, Jeju 63241, Republic of Korea

**Keywords:** silicon photomultiplier, optical fiber, plastic scintillator, radiation therapy, dosimeter

## Abstract

Existing dosimeters for radiation therapy are typically large, and their performance in in vivo system applications has not been assessed. This study develops a compact real-time dosimeter using silicon photomultipliers, plastic scintillators, and optical fibers and evaluates its in vivo applicability for radiation therapy. Dose calibration, dose-rate dependency and linearity, and short-term repeatability tests were conducted using solid water phantoms and bolus materials, and in vivo dosimetry was performed using an in-house phantom. The characterization evaluation results showed high linearity, with a coefficient of determination of 0.9995 for dose rates of 100–600 monitoring units (MU)/min, confirming an error rate within 2% when converted to dosage. In the short-term repeatability tests, the dosimeter exhibited good characteristics, with relative standard deviation (RSD) values lower than 2% for each beam delivery and an RSD value of 0.03% over ten beam deliveries. Dose measurements using the phantom indicated an average error rate of 3.83% compared to the values calculated using the treatment planning system. These results demonstrate a performance comparable to that of commercial metal-oxide-semiconductor field-effect transistors and plastic scintillator-based dosimeters. Based on these findings, the developed dosimeter has significant potential for in vivo radiation therapy applications.

## 1. Introduction

Radiotherapy involves the application of ionizing radiation, such as photons and electrons, to human tissues to destroy tumor cells [1,2,3]. The primary objective of radiotherapy is to deliver a precise radiation dose to the tumor volume while minimizing the exposure to the surrounding normal tissues [1,2,3,4,5]. Advanced techniques such as intensity-modulated radiation therapy and volumetric-modulated arc therapy have been developed and are routinely implemented in clinical practice [4,6]. However, the advancement of these techniques has introduced additional complexity into the treatment procedures, potentially increasing the risk of errors. Therefore, ensuring rigorous quality assurance of radiation therapy machines and dose delivery is essential [4]. Dosimetry has a long history of use in radiation oncology. It refers to the measurement and quantification of the effects of ionizing radiation and the process of deriving a personalized dose distribution for administration to individuals undergoing cancer radiotherapy treatment planning [7]. Accurate dosimetry methods maximize therapeutic efficacy while minimizing toxicity [1,2,3,4,5,6]. Moreover, real-time in vivo dosimetry is required to measure the dose delivered during treatment, detect significant errors, and ensure that treatments are conducted as intended [8]. Therefore, the careful selection of the appropriate dosimeter is crucial. Oversized dosimeters can experience electronic equilibrium loss, local fluence disturbance, and dose-averaging responses. In particular, a dosimeter should be user-friendly, compact, provide real-time measurements, and be water-equivalent [9].

Historically, ion chambers have been the predominant instruments for measuring absorbed doses in ionizing radiation beams, and all clinical dosimetry protocols rely on the use of ionization chambers [5]. A significant limitation of ionization chambers is their lack of portability, attributed to their considerable sizes and to the charged plates requiring substantial currents at high voltages, which necessitates the use of bulky power sources such as large batteries. The main limitation is that a large ionization chamber cannot be directly applied to a patient’s skin or incorporated into a phantom [10]. Thermoluminescence dosimetry (TLD) can provide a viable alternative for in vivo measurements [11,12]. TLD has several significant advantages such as high sensitivity, compact size, and tissue equivalence. However, TLD does not enable the real-time reading of measured results [13]. Metal-oxide-semiconductor field-effect transistor (MOSFET) devices have been used for clinical real-time in vivo dosimetry because of their excellent sensitivity, compact size, and real-time readout capabilities [14]. However, MOSFETs are expensive, have limited durability, and often exhibit short lifetimes [15,16]. A plastic scintillation optical fiber (PSOF) dosimeter can be used to quantify radiation sources [17]. A PSOF dosimeter comprises a PSOF and a photodetector [17]. A major advantage of PSOFs is that they can be fabricated entirely from plastic materials that are nearly water-equivalent [18]. In addition, PSOFs have the advantages of low cost, flexibility, and ease of production [19].

These PSOFs are sometimes used in combination with clear optical fibers [20]. Typically, clear optical fibers are unaffected by variations in temperature, pressure, and electromagnetic interference, facilitating long-distance signal transmission and real-time detection [20,21]. Owing to these advantages, optical fibers are widely employed to detect various types of radiation and physical quantities [1,9,10,18,22].

A radiation dosimeter was developed using a PSOF, optical fiber, and photomultiplier tube (PMT), and the dosimeter was characterized [1]. The results indicated good detection characteristics, up to an energy value of 20 MeV. However, the study was conducted for quality assurance purposes rather than for in vivo dosimetry. In addition, the use of PMTs introduces limitations typically associated with existing dosimeters, such as high operating voltages and large size [22,23]. Recently, silicon photomultipliers (SiPMs) have increasingly replaced PMTs owing to their broad sensitivity range (300–900 nm), high quantum efficiency (~40%), significant optical gain (∼10^7^), compact size, and immunity to magnetic field interference [23,24]. The SiPM is a type of solid-state photodetector consisting of an array of hundreds or thousands of integrated single-photon avalanche diodes (SPADs), often referred to as microcells or pixels. Each cell operates independently and is linked to a common readout system. In analog SiPMs, each cell includes a quenching resistor, and they are connected in parallel [25,26,27]. Upon detecting a photon, the SiPM generates a significant electric output signal due to internal avalanche multiplication, allowing each pixel to be counted separately either by its individual readout circuit in a digital SiPM or by measuring the amplitude (or charge) of the combined signals from the pixels in an analog SiPM. In both cases, the SiPM enables photon detection and counting with high resolution and single-photon sensitivity. The internal avalanche amplification is also fast enough to provide precise timing information about the arrival time of the detected photons, with accuracy in the range of several tens of picoseconds [25,26,27]. Common applications involving low light intensity include light detection and ranging, functional optical spectroscopy, and fluorescent light detection in both biology and physics [28]. Recently, SiPMs have also been applied in the field of medical application [29]. Berra et al. [9] developed a scintillator dosimeter using SiPMs and optical fibers to evaluate the photon beam characteristics of a linear accelerator (LINAC), including the percentage depth dose and field size. Although the detection unit was miniaturized, the overall system remained large because of the use of commercial electronics. In addition, the researchers did not assess the performance of the dosimeter as an in vivo system [9].

In this study, a PSOF dosimeter was developed using a SiPM, and the signal-processing unit was customized to miniaturize the entire system. In addition to evaluating the dosimeter characteristics, an in-house phantom was constructed to assess its potential applicability as an in vivo dosimeter. The remainder of this paper is organized as follows. Section 2 describes the development of the SiPM-based PSOF dosimeter and in-house phantom and the setup of the experimental system. In Section 3, the study results are analyzed and discussed. Section 4 presents the conclusions of this study.

## 2. Materials and Methods

### 2.1. SiPM-Based PSOF Dosimeter

**Figure 1:**
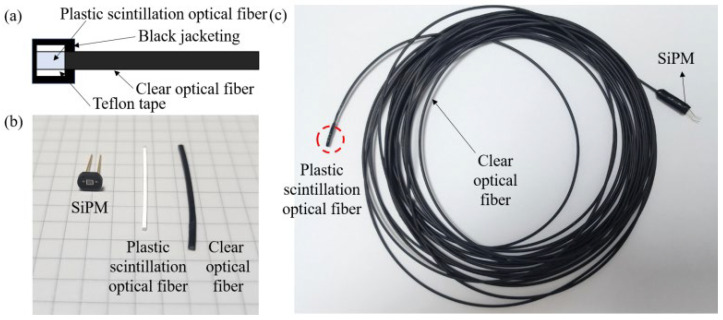
(**a**) Diagram; (**b**) materials of; (**c**) radiation detection component. The red circle is the junction between the plastic scintillation optical fiber and the clear optical fiber.

Figure 1 shows the diagram and materials of the radiation detection component and radiation detection. The radiation detection component comprises a POSF, a clear optical fiber, and a SiPM. In this study, the SCSF-78 PSOF (Kuraray, Tokyo, Japan) was selected due to the excellent alignment between its emission peak wavelength of 450 nm and the absorption wavelength of the SiPM employed in this research [30]. The PSOF had a core diameter of 1 mm and multi-cladding structure. Multi-cladding fibers exhibit an enhanced trapping efficiency, providing a higher light yield than single-cladding fibers. The decay time was 2.8 ns. In this study, the SCSF-78 PSOF was cut to a length of 2 mm, and the cut surface was polished using four different grades of polishing paper (0.3, 4, 12, and 40 µm) to improve light transmission efficiency [19]. Furthermore, a reflective layer was formed using Teflon tape to minimize light loss from the scintillating fiber, as shown in Figure 1a, and black tape was used for jacketing to shield against external light interference. The light generated by the PSOF was transmitted to the optical sensor, SiPM, through the clear optical fibers. The clear optical fibers prevented the direct exposure of the SiPM to high radiation doses while efficiently transmitting light from the scintillator to the SiPM with minimal loss. The optical fiber employed in this study is the GH4001 clear optical fiber (Mitsubishi Rayon Co., Tokyo, Japan), with a diameter of 1 mm, matching the diameter of the PSOF used in this research, and was chosen for this compatibility [31]. The optical fiber has a jacket thickness of 2.2 mm. The refractive index of the optical fiber was 1.49 [31]. The length of the clear optical fiber was set to 20 m for monitoring from the console outside the treatment room, and its surface was polished in the same way as the scintillator [32]. The SiPM used in this study was MPPC^®^ S13360-1350CS (Hamamatsu Photonics, Shizuoka, Japan). Table 1 shows the characteristics of the SiPM used in this study [32]. The effective photosensitive area of the S13360-1350CS SiPM is 1.3 mm × 1.3 mm, which is similar to the core diameter of the GH-4001 optical fiber [33]. Its spectral response range is 270–900 nm, with a peak sensitivity wavelength of 450 nm [33]. It provided a high photon detection efficiency of 40% and a gain of 1.7 × 10^6^ [33]. The breakdown voltage of this SiPM was 53.8 V, whereas the recommended operating voltage was 56.8 V. Additionally, the SiPM employed in this study features a 50 µm cell pitch. While a larger cell pitch typically results in a lower dynamic range, it offers the advantages of higher photon detection efficiency (PDE) and gain [34]. In this study, a 20 m long optical fiber was utilized. Power loss in the fiber can arise from various factors, including absorption, scattering, dispersion, bending, splicing, and impurity ions [35]. Given these considerations, a SiPM with a 50 µm cell pitch was chosen to leverage the benefits of higher PDE and gain, despite the inherent limitations in dynamic range. The interfaces between the PSOF, clear optical fiber, and SiPM were bonded using Saint-Gobain’s BC-630 optical grease. The BC-630 optical grease has a refractive index of *n* = 1.465 and a light transmission rate of approximately 95% in a wavelength range of 280 to 700 nm [22].

**Figure 2:**
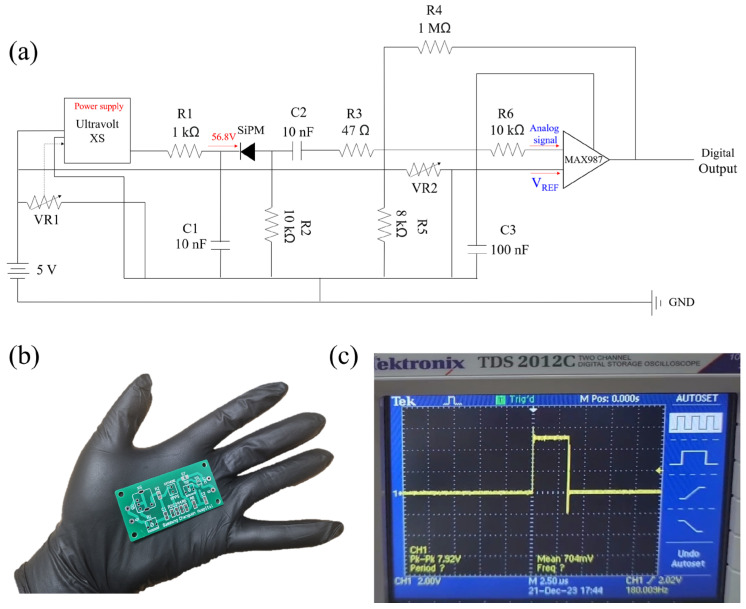
(**a**) Signal-processing circuit diagram; (**b**) circuit; (**c**) output pulse of the PSOF dosimeter.

Figure 2 shows the signal-processing circuit, circuit diagram, and output pulse of the PSOF dosimeter. In the case of the SiPM, a pulse of at least 1 V is generated in the driving circuit when radiation enters, allowing for accurate detection of radioactive rays without the need for amplification or signal formation. To reduce noise, the signal processing includes a power supply, driving circuit, and discriminator circuit (Figure 2b) [22]. The signal-processing circuit had an area of 6 cm × 3 cm. An UltraVolt^®^ XS high-voltage power supply module (Advanced Energy, Denver, CO, USA) that could deliver 0–100 V was used for the voltage supply because of its compact size, which helped minimize the overall system dimensions. The breakdown voltage of the SiPM used in this study is 53.8 V, and the recommended operating voltage is 3 V above the breakdown voltage [33]. Therefore, the supply was configured to deliver 56.8 V to the SiPM when an input of 5 V was applied. The discriminator was designed to convert signals exceeding a defined voltage threshold into 5 V digital signals, effectively filtering out noise from the actuation circuit and ensuring that only radiation signals were transmitted to the microcontroller unit (MCU). A MAX987 comparator (Analog Devices, Inc., Wilmington, MA, USA) was employed to design the discriminator. The V_THR_ (threshold voltage) for noise removal can be calculated using the following equation [36]:

(1) $$V_{THR}=\left[\frac{R4+R5+R6}{R4\times R5}\right]\times V_{REF}.$$

Here, V_REF_ is the reference voltage, which can be adjusted using the VR2 (variable resistor). In this study, the V_THR_ was adjusted to ensure that the count/s were zero in the absence of radiation. The MCU used in this study was an Arduino Uno (Arduino LLC, Boston, MA, USA) based on an Atmega328 microcontroller (Atmel, San Josem, CA, USA). The Arduino board processed the digital output signals from the POSF dosimeter, counted the radiation events, and displayed the results using a PC interface.

### 2.2. In-House Phantom

**Figure 3:**
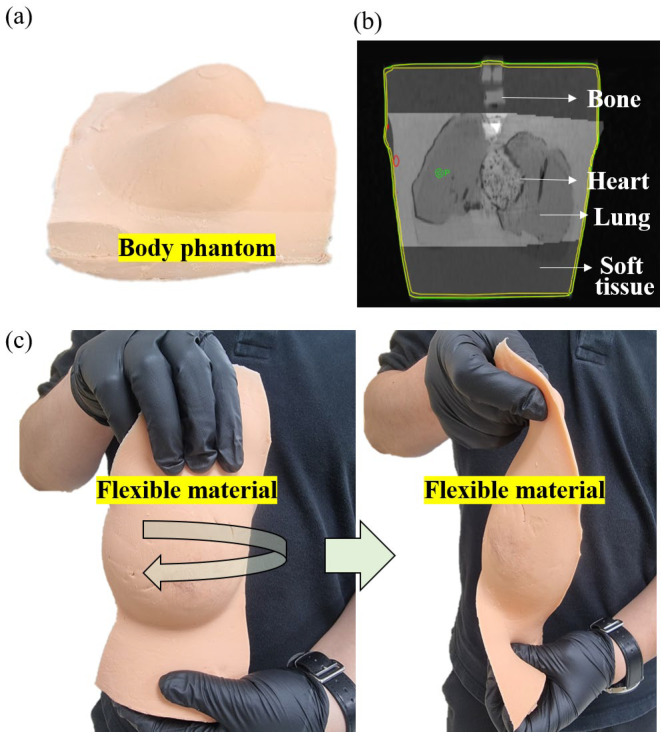
(**a**) In-house phantom; (**b**) image from its CBCT; (**c**) soft-tissue material.

In in vivo dosimetry measurements, a human-like phantom may be used instead of a person [37]. However, in the case of the commercial phantom, devices such as optically stimulated luminescent dosimeters (OSLDs) are designed for insertion, while other dosimeters are not intended for insertion. In this study, a phantom that mimicked the human skin was developed to evaluate the performance of the fabricated PSOF dosimeter. Figure 3 shows the in-house phantom, an image from the cone beam computed tomography (CBCT) of the in-house phantom, and a soft-tissue material. The soft-tissue component was made of EcoFlex™ 0010 (Smooth-On Inc., Macungie, PA, USA), a silicone rubber material. With a shore hardness of 0.1, this material is exceptionally soft, making it suitable for simulating soft tissues (Figure 3c) and safe for skin contact [38]. The EcoFlex™ 0010 material consisted of a base and a curing agent at a mixing ratio of 1:1. After mixing the base and curing agent, the mixture was poured into a human-body-model mold to create a soft-tissue structure. The working time was 30 min and the curing time was 4 h. The bones were constructed using gypsum sheets (Nok-San Cast, Chungju, Republic of Korea). Water was applied to the gypsum sheet, which was then adhered to a bone-shaped plastic mold to form the bone structure. The artificial lung was made of polyurethane rubber, specifically VytaFlex™ 10 (Smooth-On Inc., Macungie, PA, USA) with a shore hardness of 10. VytaFlex™ 10 comprised a base and a curing agent, with a mixing ratio of 1:1. After mixing the base and curing agent, the mixture was poured into a lung-model mold to construct a lung structure. The working time was 30 min, and the curing time was 24 h. The heart was fabricated using alginate, known as Alja-Safe™ (Smooth-On Inc., Macungie, PA, USA). Alja-Safe™ was mixed with water at a 1:1 ratio and poured into the heart-model mold. The working and curing times were 5 and 8 min, respectively. Computed tomography imaging of the fabricated body phantom was performed using a Discovery CT590 RT simulator (GE Healthcare, Milwaukee, WI, USA) for treatment planning. The reconstructed computed tomography images had a slice thickness of 2.5 mm.

### 2.3. Experimental Setup

**Figure 4:**
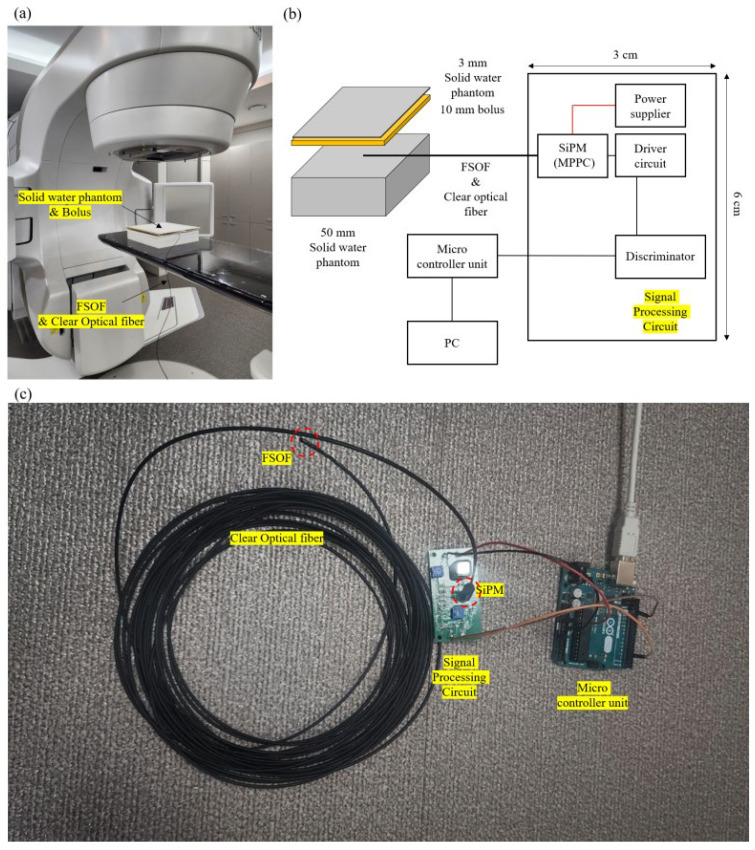
(**a**) Experimental setup, (**b**) system schematic diagram, and (**c**) PSOF dosimeter for dose calibration and reproducibility test.

Figure 4 shows the experimental setup for the dose calibration, linearity, and reproducibility tests. The calibration and reproducibility tests of the POSF dosimeter were performed using a TrueBeam STx LINAC (Varian Medical System, Palo Alto, CA, USA) with a photon energy of 6 MV, source-to-surface distance of 100 cm, dose maximum depth (d_max_) of 1.3 cm, and field size of 10 cm × 10 cm. One hundred monitoring units (MU) were administered along with water-equivalent materials of appropriate thickness, such as a solid water phantom and bolus, to achieve a nominal dose of 1 Gy at the detector (Figure 4a). The dose per MU was calibrated using a cylindrical ion chamber, traceable to an accredited calibration laboratory, in accordance with the International Atomic Energy Agency’s (IAEA) Technical Reports Series (TRS) 398 protocol, ensuring that 1 MU corresponds to 1 cGy under the specified irradiation conditions [39]. TRS-398 is a code of practice for determining the absorbed dose in external beam radiotherapy. The formula for calculating the dose per MU in accordance with the TRS-398 protocol is as follows [40]:

(2) $$D_{W,Q}\left(Z_{ref}\right)=M_{Q}N_{D,W,Q_{0}}k_{{Q,Q}_{0}}$$

The absorbed dose to water at the reference depth, $Z_{ref}$, in a water phantom irradiated by a beam of quality Q is denoted as $D_{W,Q}\left(Z_{ref}\right)$. M_Q_ represents the dosimeter reading under reference conditions, which must be corrected for factors such as polarity and recombination effects. The calibration factor, $N_{D,W,Q_{0}}$, represents the absorbed dose to water for the dosimeter and is determined by a standard laboratory at the reference beam quality. If the dosimeter is used with a beam quality Q different from the calibration beam quality Q_0_, the absorbed dose to water must be corrected using a beam quality correction factor to account for the difference between the reference beam quality Q_0_ and the actual user beam quality Q. $k_{{Q,Q}_{0}}$ is a factor used to adjust for the difference in the response of an ionization chamber between the reference beam quality Q_0_, used for calibrating the chamber, and the actual user beam quality Q [40].

The dose rates used for calibration ranged from 100 to 600 MU/min at intervals of 100 MU/min. In the short-term repeatability tests, the dose rate was fixed at 400 MU/min, and 100 MU was delivered in 10 repetitions. Linearity and repeatability were evaluated using the coefficient of determination (R^2^) and the relative standard deviation (RSD), respectively [23]. R^2^ was calculated by fitting the correlation between the dose rate and the mean count rate, whereas RSD was calculated as follows [39]:

(3) $$RSD\left(\%\right)=\left[\frac{{\left\{\sum {\left(X_{i}-X_{ave}\right)}^{2}/n\right\}}^{0.5}}{X_{ave}}\right]\times 100.$$

In Equation (3), $X_{i}$ is the individual measured value, $X_{ave}$ is the average of all measured values, and *n* is the total number of measurements.

**Figure 5:**
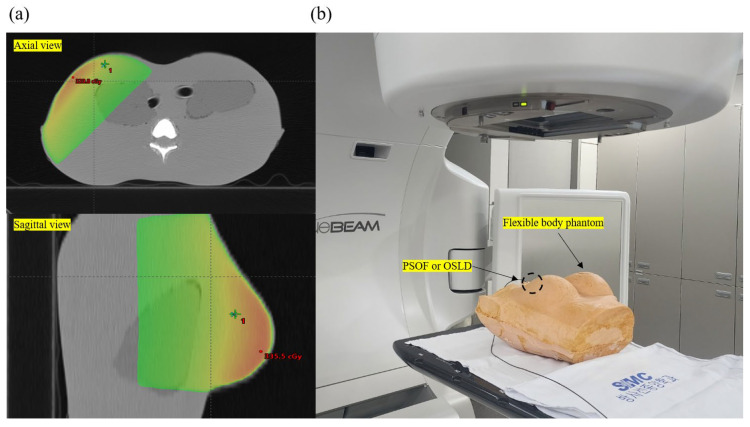
(**a**) Dose distribution of three-dimensional conformal radiotherapy treatment plan; (**b**) experimental setup.

Figure 5a shows the dose distribution of the treatment plan performed on the in-house phantom and the experimental setup for dose evaluation of the PSOF dosimeter following the treatment plan. Three-dimensional conformal radiotherapy treatment (3D CRT) was planned using the Eclipse Treatment Planning System (TPS), version 16 (Varian Medical System, Palo Alto, CA, USA). An anisotropic analytical algorithm and a photon optimizer algorithm were used. In the 3D CRT, parallel-opposed tangential fields were employed using a 6 MV photon beam. The maximum dose rate was 400 MU/min. A dynamic wedge was used at an angle of 45°. The planning target volume included the entire right breast, and the prescribed dose was 3 Gy in one fraction. In vivo measurements were conducted, as shown in Figure 5b, with measurement points located 8 cm from the left side of the phantom and 11 cm from top to bottom at a height of 15 cm. The measurements were performed using the PSOF dosimeter developed in this study and an InLight^®^nanoDot™ OSLD (Landauer^®^, Glenwood, IL, USA). The TPS doses were compared based on the error rate, which was calculated by dividing the difference between the calculated and measured TPS values by the calculated TPS value [41]. The PSOF dosimeter acquired detection values in real time. In contrast, OSLD detection values were obtained 2 h later using the microSTAR^®^ reader system (Landauer^®^, Glenwood, IL, USA). Measurements were performed in triplicates for the dosimeter.

## 3. Results and Discussion

**Figure 6:**
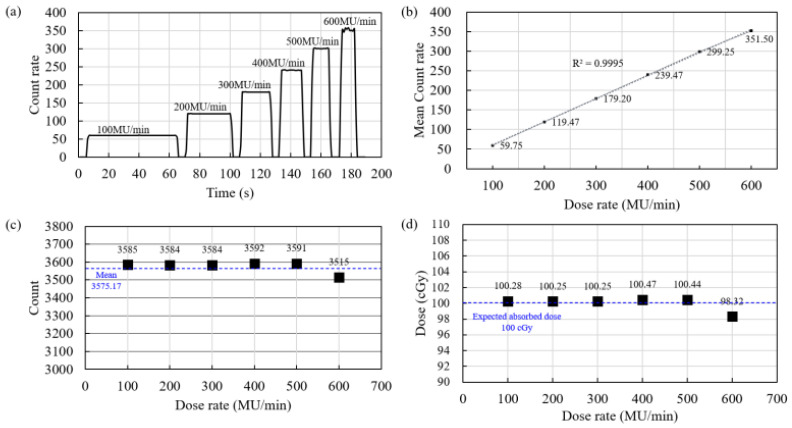
Variations in various dosimeter-measured parameters with dose rate: (**a**) count/s; (**b**) mean count/s; (**c**) total count; (**d**) absorbed dose.

Figure 6a shows the raw data, and Figure 6b shows the mean count rate (count per sec) at each dose rate, along with a linear fitting of the mean count rate. The linear fitting showed an R^2^ value of 0.9995, demonstrating that the measurements exhibited a highly linear relationship with the dose rate. Figure 6c shows the total counts measured at each dose rate. The mean total count was 3575.17, and as 100 MU was delivered at each dose rate, the mean count per MU was 35.75. Because the experiments were conducted under reference conditions (source-to-surface distance: 100 cm; field size: 10 cm × 10 cm; d_max_: 1.3 cm), a value of 35.75 counts could be converted to 1 cGy. Figure 6d shows the absorbed dose values converted from the total counts measured at each dose rate using the calibration factor. A comparison between the converted and expected values at each dose rate indicated a mean error factor of 0.56%. Moreover, the maximum error factor was 1.68% when 100 MU was delivered at 600 MU/min. These results indicate that the dose-rate dependency was within 2%. A dosimeter should be unaffected by the dose rate. Ferrer et al. [41] characterized a commercial plastic scintillator dosimeter using Unity (Elekta, Stockholm, Sweden), a type of magnetic resonance, (MR)-LINAC. In their study, the dose rate was varied from 43 to 430 MU/min to assess its dependence on dose, and the results showed an average variation of 0.35% and a maximum variation of 1.19% [41]. Kumar et al. [42] observed that a commercial MOSFET dosimeter exhibited a dose-rate dependency of ±2% within the range of 100–600 MU/min. Therefore, the dosimeter developed in our study exhibited a performance comparable to that of commercial dosimeters based on the dose-rate dependency results.

**Figure 7:**
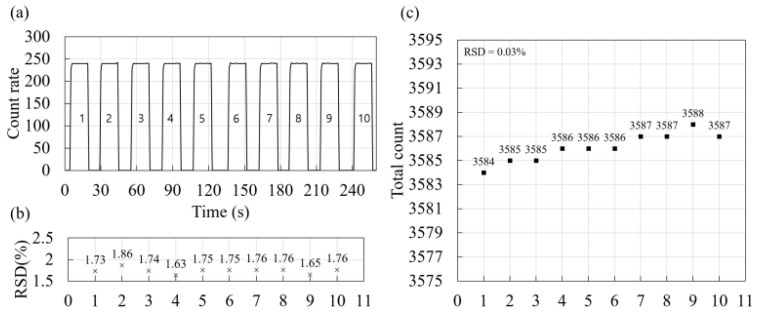
(**a**) Raw data; (**b**) RSD of raw data; (**c**) total counts of the dosimeter from short-term repeatability tests.

Figure 7a shows the raw data obtained using the dosimeter during the short-term repeatability tests, and Figure 7b presents the RSD values for each irradiation. The average RSD was 1.74%, with a maximum value of 1.86%. This indicated that the variability in the measured values within a single irradiation was minimal. Increasing the dose at a constant rate was equivalent to extending the irradiation time. Given the minimal variability observed within a single irradiation, accurate measurements can be maintained, even when assessing higher or lower doses. Figure 7c shows the total count values measured during the short-term repeatability tests and their corresponding RSD values. The average total count was 3585.33, with a standard deviation of 1.2 and an RSD value of 0.03%. These results demonstrate the excellent repeatability of the developed dosimeter. Based on the results of short-term repeatability tests for a commercial plastic scintillator dosimeter, Ferrer et al. [43] found that the variation was lower than 0.4% after delivering 100 MU ten times. This suggests that the dosimeter developed in our study exhibits performance comparable to that of a commercial plastic scintillator dosimeter.

Table 2 presents the in vivo measurements obtained with the PSOF and OSLDs. The error rate was calculated by subtracting the measured value from the expected value, dividing the result by the expected value, and then multiplying by 100. The developed dosimeter showed an average error rate of 3.83% and a maximum error rate of 8.83% compared with the doses calculated using the TPS. When measured at the same points as the commercial OSLD, an average error rate of 2.07% and a maximum error rate of 6.18% were obtained. These results indicate that the developed dosimeter exhibited comparable performance to a commercial OSLD, while also enabling real-time dose measurements. The RSD values for the POSF dosimeter and OSLD dosimeters were 6.36% and 2.53%, respectively. This indicates that the variability of the POSF dosimeter was higher than that of the OSLD. However, this trend might have occurred because a hole was created in the in-house phantom to insert the dosimeter, which was sized according to a larger-volume OSLD. Consequently, the smaller-volume POSF dosimeter might have shifted within the hole during the measurement process. In this study, challenges were encountered when inserting optical fibers into a commercial anthropomorphic phantom, leading to the development of an in-house phantom. The fabricated phantom was used to position the setup using imaging-guided techniques. Various materials with different HU values, such as silicone, polyurethane, and alginate, were used to simulate the organs to improve the accuracy of the positioning setup. However, as indicated by the dose measurement results, limitations were encountered in stabilizing the dosimeter position. The developed dosimeter uses a plastic scintillator as its detection component; thus, it is water-equivalent. Moreover, the use of optical fibers is not affected by environmental factors, such as temperature and pressure, facilitating real-time remote measurements [44]. In addition, the proposed dosimeter has a significant advantage owing to its compact size. In several studies, only a detection unit was developed, whereas the signal-processing unit relied on commercial components, thereby limiting the potential for dosimeter miniaturization [1,9]. The commercial dosimeter used by Ferrer et al. [45] was the mobile MOSFET dosimeter from Best™ Medical Canada (Ontario, Canada), with a data reader size of 17.8 cm × 15.9 cm × 4.2 cm. By comparison, the data reader developed in this study had a signal-processing unit with dimensions of 6 cm × 3 cm × 1.3 cm and an MCU of 6.8 cm × 5.8 cm × 1.2 cm. When combined, the maximum size was 6.8 cm × 5.8 cm × 2.5 cm, indicating a considerably more compact form factor than that of the commercial dosimeter reader. This demonstrates that the developed dosimeter satisfied all the essential requirements, as outlined in the introduction section [9]. Furthermore, the developed dosimeter showed a low dependence on the dose rate, exhibited high short-term repeatability, and demonstrated high accuracy in in vivo dose measurements using a phantom. Therefore, it is suitable as an in vivo dosimeter.

## 4. Conclusions

In this study, a SiPM, optical fibers, plastic scintillators, and a compact electronic system were used to develop an in vivo dosimeter for radiation therapy, and the dosimeter characteristics were evaluated. The characterization analysis results demonstrated that the developed dosimeter exhibited high linearity with respect to the dose rate and low dependency on the dose rate, comparable to that of commercial MOSFET and plastic scintillator-based dosimeters. Furthermore, the short-term repeatability characteristics were closely aligned with those of plastic scintillator-based dosimeters. The absorbed dose values obtained using the phantom indicated minimal differences between the calculated values obtained using the TPS and the measured values. Based on these findings, the developed dosimeter has a significant potential for in vivo radiation therapy applications. In future research, holders or devices that can accurately position the dosimeter and improve the positional accuracy of the phantom should be developed to enhance dose measurement accuracy.

## Figures and Tables

**Table 1:** Characteristics of S13360-1350CS [33].

Model	S13360-1350CS
Package type	Ceramic
Effective photosensitive area	1.3 mm × 1.3 mm
Number of pixels	667
Pixel size	50 µm
Spectral response rang	270 to 900 nm
Peak sensitivity wavelength	450 nm
Gain	1.7 × 10^6^
Dark count (Typ.)	90 kcps
Dark count (Max.)	270 kcps
Recommended operating voltage	Breakdown voltage + 3 V

**Table 2:** In vivo measurements obtained with the PSOF and OSLDs.

Dosimeter	Measument 1 (cGy)	Measument 2 (cGy)	Measument 3 (cGy)	Mean (cGy)	RSD (%)
PSOF	294.19	338.78	336.72	323.23	6.36
OSLD	295.13	310.31	309.18	304.87	2.53

## Data Availability

Data is contained within the article.
